# A comparison of proteomic, genomic, and osteological methods of archaeological sex estimation

**DOI:** 10.1038/s41598-020-68550-w

**Published:** 2020-07-17

**Authors:** Tammy Buonasera, Jelmer Eerkens, Alida de Flamingh, Laurel Engbring, Julia Yip, Hongjie Li, Randall Haas, Diane DiGiuseppe, Dave Grant, Michelle Salemi, Charlene Nijmeh, Monica Arellano, Alan Leventhal, Brett Phinney, Brian F. Byrd, Ripan S. Malhi, Glendon Parker

**Affiliations:** 1 Department of Environmental Toxicology, University of California, Rm 5241B Meyer Hall, 1 Shields Ave, Davis, CA 95616 USA; 2 Department of Anthropology, University of California, Davis, USA; 3 Program in Ecology, Evolution and Conservation Biology, University of Illinois, Urbana-Champaign, USA; 4 Far Western Anthropological Research Group, Inc, Davis, CA USA; 5 Department of Anthropology, University of Illinois, Urbana-Champaign, USA; 6 D&D Osteological Services, LLC, San Jose, CA USA; 7 Proteomic Core Facility, Genome Center, University of California, Davis, CA USA; 8 Muwekma Ohlone Tribe of the San Francisco Bay Area, Milpitas, CA USA; 9 Department of Anthropology, San Jose State University, San Jose, CA USA; 10 Carl R. Woese Institute for Genomic Biology, University of Illinois, Urbana-Champaign, USA

**Keywords:** Proteomics, Mass spectrometry, Archaeology, Genomics, Biological anthropology

## Abstract

Sex estimation of skeletons is fundamental to many archaeological studies. Currently, three approaches are available to estimate sex–osteology, genomics, or proteomics, but little is known about the relative reliability of these methods in applied settings. We present matching osteological, shotgun-genomic, and proteomic data to estimate the sex of 55 individuals, each with an independent radiocarbon date between 2,440 and 100 cal BP, from two ancestral Ohlone sites in Central California. Sex estimation was possible in 100% of this burial sample using proteomics, in 91% using genomics, and in 51% using osteology. Agreement between the methods was high, however conflicts did occur. Genomic sex estimates were 100% consistent with proteomic and osteological estimates when DNA reads were above 100,000 total sequences. However, more than half the samples had DNA read numbers below this threshold, producing high rates of conflict with osteological and proteomic data where nine out of twenty conditional DNA sex estimates conflicted with proteomics. While the DNA signal decreased by an order of magnitude in the older burial samples, there was no decrease in proteomic signal. We conclude that proteomics provides an important complement to osteological and shotgun-genomic sex estimation.

## Introduction

Biological sex plays an important role in the human experience, correlating to lifespan, reproduction, and a wide range of other biological factors^1–5^. Sex and gender are also fundamental in structuring an array of cultural behaviors, including residence patterns, kinship, economic roles, and identity construction and expression^6–9.^ How sex interacts with gender and these particular issues is not static and can vary in detail across societies and over time^10–12^. It is not surprising that sex is one of the most basic and important measures in bioarchaeological and forensic analyses.

Typically, osteological features are used to estimate sex of skeletal remains, and the most widely used marker is the morphology of the *os coxae*^13–16^. However, appropriate markers are not always sufficiently expressed or preserved to estimate sex using morphological criteria^17^. A lack of sexually-dimorphic markers is especially acute for skeletons of infants and children who have not undergone puberty. Mortuary practices, such as cremation or secondary burial in charnel houses, can also can impose limitations on the utility of osteological sex estimates^18^.

The advent of DNA sequencing made it possible to use skeletal remains to estimate the sex of very young individuals; it also expanded sex estimations for fragmentary, pathological, and degraded skeletal materials^19–21^. More recently, development of massively parallel DNA sequencing greatly improved genome coverage in archaeological samples^22–25^. In addition to providing detailed genetic information, this allows biological sex to be estimated from shotgun sequencing data^25–27^. These approaches were an improvement over earlier PCR-based marker methods, which were less sensitive and had a higher risk of contamination^28–32^. Even with the application of high-throughput genomic data, confident estimation of biological sex is still restricted by requirements for high levels of DNA preservation^27^.

Recently, proteomic analysis of sex-specific amelogenin peptides in tooth enamel has been forwarded as an additional means of sex estimation in archaeological settings^33–37^. Amelogenin genes are well-studied genetic markers of the X and Y chromosomes and have long been a basis of forensic sex determination^20,38–40^. Proteins can be useful targets for analysis in many archaeological settings as their molecular structure is more favorable for preservation relative to DNA^41–44^. Moreover, because amelogenin peptides are incorporated within the mineral phase of tooth enamel, the hardest and most durable material in the human body, such peptides may be particularly stable and persistent over long periods of time^45–47^.

**Figure 1:**
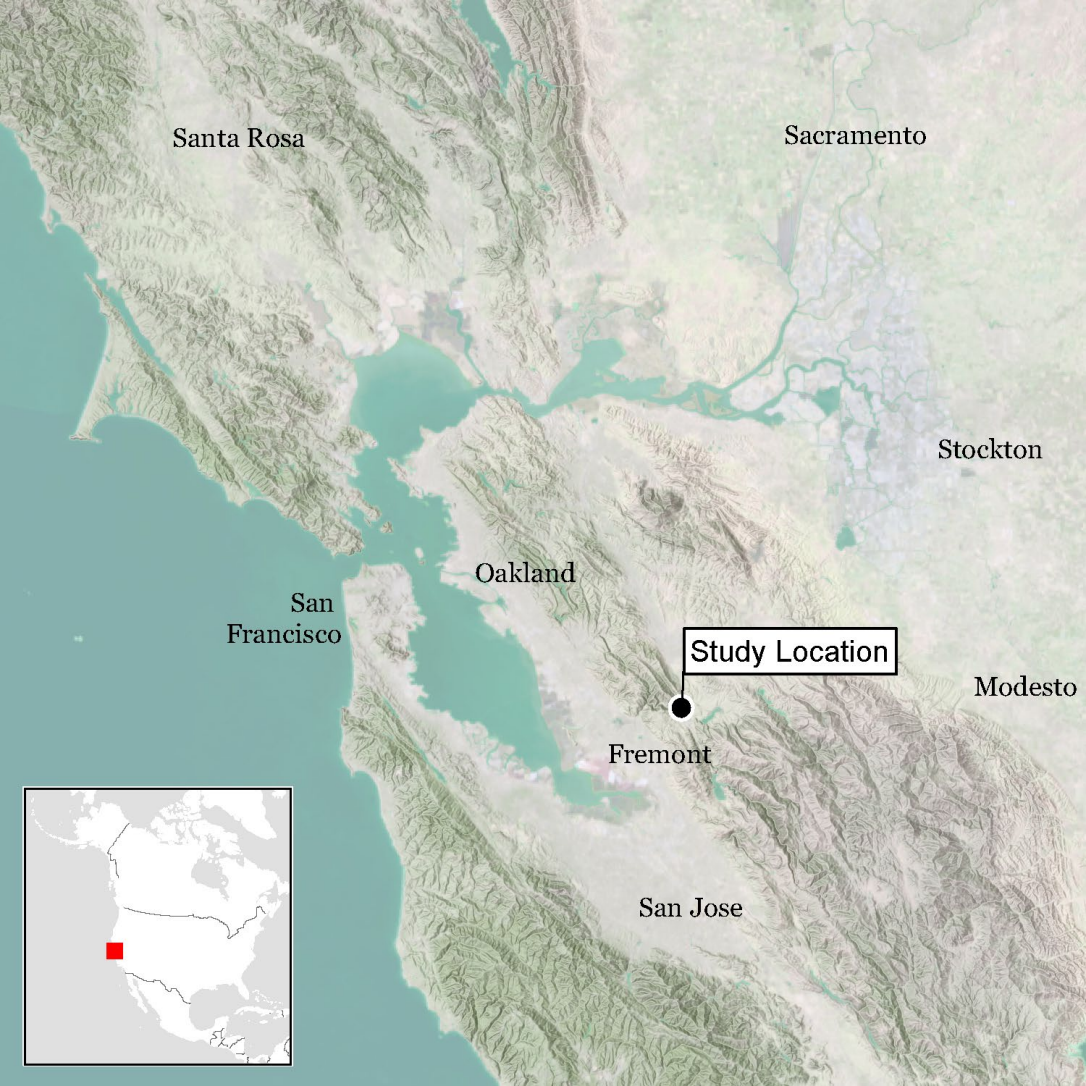
Map showing general location of *Síi Túupentak* (CA-ALA-565/H) and *Rummey Ta Kuččuwiš Tiprectak* (CA-ALA704/H) in the San Francisco Bay area of California. Map was created by Far Western Anthropological Research Group with ESRI ArcGIS Desktop 10.6 (https://www.esri.com/).

The availability of three independent methods of sex estimation provides an opportunity to compare and cross-check techniques against one another. While recent remains of known sex can be used to validate and estimate the precision of these techniques, such remains do not replicate archaeological conditions. In the current study, we apply three techniques: proteomic analysis of amelogenin peptides, shotgun-sequenced DNA, and standard osteological methods to determine the sex of human remains from two Late Holocene ancestral Ohlone villages in Central California: *Síi Túupentak* (CA-ALA-565/H; ca. 600–100 cal BP) and *Rummey Ta Kuččuwiš Tiprectak* (CA-ALA-704/H; ca. 2,440–180 cal. BP) (Fig. 1). Genomic data were further analyzed using two distinct algorithms, one that compared the ratio of Y-chromosome reads to all sex chromosome reads (R_Y_)^48^, and another that compared the ratio of X-chromosome reads to all autosomal reads (R_X_)^27^. In many cases (n = 55) each method of sex estimation, genomic, proteomic, and osteological, was applied to remains from the same individual.

To date, this is the largest study using sexually dimorphic amelogenin peptides to estimate biological sex and the largest to estimate sex based on matching shotgun DNA sequencing^25,27^. This allows us to directly compare the respective techniques at a statistical level, and provides a broader framework for interpretation of sex estimation data that employs the strengths and limitations of each approach.

## Background

### Genomic methods for sex estimation

Earlier PCR-based approaches that targeted sex-specific molecular markers, usually the amelogenin gene family, were often affected by modern contamination^20,30,32^. A benefit of shotgun DNA sequencing is that it can detect chemical modifications characteristic of ancient DNA (aDNA) and identify exogenous DNA contamination^49^. Skoglund and colleagues^25^ developed a genomic method of sex determination that takes advantage of high-throughput shotgun-DNA sequencing. This method (R_Y_) estimates sex using sequence reads of 30 base pairs (bp) or longer that map to human X- and Y-chromosomes. R_Y_ is calculated as the number of Y-mapped reads compared to the total number of X- and Y- mapped reads. The R_Y_ method does not filter out homologous portions, but relies on a large number of total sequences to return a robust determination of sex. R_Y_ criteria were defined based on published data from 14 modern humans of known sex and 16 archaeological remains that had high-quality, prior PCR-based sex determinations. By artificially down-sampling sequences from these same individuals, Skoglund et al.^25^ recommended that a minimum of 100,000 total chromosome reads mapped to the human reference genome (or 3,000 reads mapped to sex-chromosomes) were needed for confident sex estimations.

This degree of preservation may be problematic for many archaeological remains, as noted by Mittnik et al.^27^. To reduce the required number of mapped human sequences, Mittnik and colleagues proposed an alternative method of sex estimation (R_X_) using high-throughput shotgun-sequenced DNA. The R_X_ method relies on the proportion of reads mapped to the human X chromosome compared to the proportion of reads mapped to each of the autosomal chromosomes. By down-sampling reads from the same high-quality ancient DNA data sets used in Skoglund et al.^25^, the R_X_ method was able to give confident assignments with as few as 1,000 human genome reads^27^.

### Proteomic approach to sex estimation

Amelogenin genes are located on both the X- and Y- chromosomes in humans and play a major role in the biosynthesis of enamel^50–52^. These genes express distinctive isoforms of amelogenin proteins, AMELX_HUMAN (AMELX) and AMELY_HUMAN (AMELY)^38,40,53^, and detection of these proteins can be used to estimate sex over archaeological time scales^33–35,37,54,55^. Nano-liquid chromatography coupled with orbitrap tandem mass spectrometry (nLC-MS/MS) allows peptides to be identified at two levels. The MS1 level measures the precise molecular mass of the intact peptide, and subsequent MS2 data results in a spectrum of fragmented masses that together can be used to statistically match the most likely amino acid sequence to mass fragments of the MS1 peptide^56^. Signals from peptides with unique amino acid sequences specific to either AMELX or AMELY are identified, while those that are homologous are filtered out. Following removal of these non-specific amelogenin peptides, signals of all peptides unambiguously attributed to either AMELX or AMELY are then combined into a single measure^33^. This process differs from the methods of Stewart et al.^34,36,57^, Wasinger et al.^35^, or Froment et al.^54^, which relied on detection of two or four unique peptide masses only. In contrast, the proteomic method employed here identifies and sums signal intensities of multiple different AMELX and AMELY peptides with various permutations of common post translational modifications (PTMs), such as deamidation or oxidation. The ability to measure a greater number of specific peptides should increase sensitivity. Sensitivity is also likely to be increased in our method by using destructive chemistries as opposed to simple acid-leaching that seeks to preserve gross anatomy^34,58^.

### Archaeological sites

*Síi Túupentak* (CA-ALA-565/H) and *Rummey Ta Kuččuwiš Tiprectak* (CA-ALA704/H*)* are ancestral Native American Ohlone settlements situated in a well-watered valley in the southeast portion of the San Francisco Bay region, Central California, USA (Fig. 1). Large-scale infrastructure construction required substantive archaeological excavations at both sites, which were carried out by the Far Western Anthropological Research Group (FWARG)^59,60^. Prior to fieldwork, the state-appointed Most Likely Descendent of the Muwekma Ohlone Tribe recommended detailed analysis of all ancestral remains encountered. The Tribe collaborated with FWARG on the project, participated in all aspects of fieldwork, and were the primary excavators of all burials. Tribal leadership approved all analytical studies of ancestral remains and partnered with the research team to conduct this research. All burials were subject to osteological analysis (n = 105), all radiocarbon-dated burials (n = 99) were sampled for DNA, and 55 were sampled for amelogenin proteins. Archaeological mitigation of construction impacts to these archaeological sites, including the discovery, excavation, analysis and reporting of human remains, strictly conformed to all state and local laws and regulations. Members of the Muwekma Ohlone have seen and been provided an opportunity to contribute to the final version of the write-up of this study. In addition to their contributions to this study, the Muwekma Ohlone have advocated for science and genomics as a tool for Indigenous peoples and have strongly supported the Summer internship for INdigenous peoples in Genomics (SING) program.

## Results

### Archaeological contexts

*Síi Túupentak* (CA-ALA-565/H) is a large, intensively occupied Late Period village (129 radiocarbon dates from features, burials, and site midden range from 605–100 cal BP) with both domestic debris and associated cemetery^59^. Sixty-six burials, comprised of 76 individuals, were recovered. Most (71%) were primary inhumations, along with 21% secondary cremations, and 8% secondary inhumations. The extent and degree of burning varied between cremations, with the vertebrae, sacrum, pelvis, and proximal femora being the most commonly preserved elements. Dentition was generally not present for cremations but those with suitable preservation were analyzed. Seventy burials were dated between 600 to 110 cal BP (1,350 to 1,840 CE). Excluding two outliers, most date to a 345-year time span from 525 to 180 cal BP (1,425 to 1,770 CE). All dates were calibrated with a mixed marine curve based on established protocols using individual δ^13^C values, and median intercepts were used to organize the results^61^. In contrast, nearby *Rummey Ta Kuččuwiš Tiprectak* (CA-ALA704/H*)* is a substantial multicomponent settlement with occupation ranging from 2,440–175 cal BP (490 BCE–1775 CE), based on 60 radiocarbon dates from generalized site deposits, features, and burials^60^. With 88% of dates falling between 2,440–1,610 cal BP, occupation was most intensive in the Early/Middle Transition (2,650–2,150 cal BP) and Middle 1 periods (2,150–1,530 cal BP)^59,60^. Twenty-five burials comprising 29 individuals were recovered. Virtually all (93%) were primary inhumations, with just 7% secondary inhumations. Most interments (n = 26) date from 2,240–1,610 cal BP (290 BCE–340 CE, a 630-year span primarily in the Middle 1 period), but three date later in time, including two that are contemporary with Late Period *Síi Túupentak* (Fig. 1).

### Sensitivity of different methods

Data for all burials recovered from both sites (n = 105) is available in supplemental materials (Table S1). Table S2 shows results of the 55 samples from CA-ALA-565/H and CA-ALA-704/H where analysis by each of the three methods was attempted. Proteomic analysis of amelogenin provided sex estimates in all 55 cases (100%). DNA shotgun sequencing produced reads that mapped to the human reference genome for 53 of the 55 samples (96%). Genomic sex estimation using the ratio of Y chromosome reads compared to total sex chromosome reads (R_Y_) provided 43 sex estimates (78%). The R_X_ method, that compared the ratio of reads mapping to the X-chromosome with those mapping to each autosomal chromosome, resulted in 50 sex estimates (91%). Osteology provided sex estimates for 28 of the 55 common samples (51%).

Sex estimates fell into definitive or conditional categories. All proteomic estimates were definitive in this study, with all males having more than two unique AMELY_HUMAN (AMELY) peptides and females having a probability of female sex (Pr(F)) greater than 0.5 (Methods)^33,62,63^. DNA-based conditional, or “consistent with . . .”, estimates had 95% confidence intervals for the ratios that crossed thresholds for definitive XX or XY karyotype assignment^25,27^. Indeterminate samples fell entirely between the two thresholds. Using the genomic R_Y_ method, 27 sex estimates (49%) were definitive and 16 were conditional (21%). For the R_X_ method, 26 estimates were definitive (47%) and 24 were conditional (44%). For osteology, conditional estimates were assigned as either “probable”, or “possible”, with the latter having less certainty. Osteology provided 15 definitive estimates (27%) and 13 conditional (24%) estimates (Table S2).

### Comparison of genomic and proteomic sex estimation

Overall, there was high consistency between the methods. Table 1 shows pair-wise comparisons of the proportion of total agreements and disagreements in sex estimates for both definitive and conditional estimates between each method. Proteomic estimates agreed with osteological estimates in 27 of 28 cases (96%, Table 1). Genomic estimates using the R_Y_ method agreed with osteological estimates in 18 of 23 cases (82%), and in 20 out of 25 cases (80%) using the R_X_ method. Genomic estimates agreed with proteomic sex estimates in 36 of 43 cases (84%) using the R_Y_, and 41 out of 50 cases (82%) when using the R_X_ method (Table 1).

**Table 1:** Comparisons of consistant, inconsistent definitive, and inconsistent conditional sex estimates across proteomic, genomic, and osteological methods.

	RY	RX	Prot
**Total consistent sex estimates**			
Oste	78% (18/22)	80% (20/25)	96% (27/28)
RY		98% (42/43)	84% (36/43)
RX			82% (41/50)
**Inconsistent definitive sex estimates**			
Oste	0% (0/8)	0% (0/7)	0% (0/15)
RY		0% (0/18)	7% (2/27)
**RX**			0% (0/26)
**Inconsistent conditional sex estimates**			
Oste	29% (4/14)	28% (5/18)	8% (1/13)
RY		4% (1/25)	31% (5/16)
RX			38% (9/24)

All proteomic estimates were definitive in this study. For osteology, conditional estimates were assigned as either “probable”, or “possible”, with the latter having less certainty. DNA-based conditional estimates (“consistent with XX, but not XY” or, “consistent with XY, but not XX”) have 95% confidence intervals that cross thresholds for definitive XX or XY karyotype assignment.

A closer look at differences between the genomic and proteomic methods is instructive. Although the proteomic method was able to estimate sex in all cases, several were indeterminate using genomic methods, with the R_Y_ and R_X_ method unable to estimate sex in 12 and 5 cases respectively. Two of the cases were indeterminate because DNA extraction and sequencing was not successful, while remaining cases were indeterminate based on their calculated values (Tables S2, S3).

In addition to the indeterminate cases described above, there were inconsistencies between genomic-based and proteomics-based estimates (Tables 1 and 2). On two occasions definitive sex estimates based on R_Y_ values were inconsistent with proteomic sex estimation (CA-ALA-565/H Burial 5A and CA-ALA-704/H Burial 23, Tables 2, S1, S2, S3). There were no inconsistencies with definitive R_X_ sex estimates. Proteomic sex estimation resulted in a different sex assignment than conditional DNA estimates for 9 out of 24 (38%), and 5 out of 16 (31%) individuals when the R_X_ and R_Y_ ratios were used, respectively (Table 1).

**Table 2:** List of sex estimations by increasing number of matched human DNA sequences. Conditional sex estimates are indicated with an asterisk.

Site and Burial	N DNA seq	Oste	R_Y_	R_X_	Prot
ST-30	0	Indet	Indet	Indet	M
ST-38	0	Indet	Indet	Indet	M
**RTKT-5**	202	M*	Indet	Indet	M
**RTKT-21**	384	Indet	Indet	F*	F
**RTKT-4**	602	M*	Indet	Indet	M
**RTKT-3**	614	M*	Indet	F*	M
**RTKT-14**	884	Indet	Indet	F*	F
**RTKT-22**	1,499	M*	Indet	M*	M
**RTKT-7C**	2036	Indet	F*	M*	F
**RTKT-8**	2,249	Indet	Indet	M*	M
ST-21	3,838	Indet	F*	F*	M
ST-55	3,940	Indet	F*	F*	F
ST-8	5,256	Indet	M*	M	M
ST-5A	6,605	Indet	F	F*	M
ST-49B	8,171	M	M*	M	M
ST-31	8,491	F*	F*	F*	F
ST-29	12,880	F*	M*	M*	F
**RTKT-12**	16,208	Indet	M*	M	M
ST-13	17,650	M	M*	M	M
ST-35	19,768	M	F*	F*	M
ST-58B	23,515	Indet	F*	F*	F
ST-23	24,618	Indet	F	F*	F
**RTKT-19**	33,738	Indet	M*	M	M
ST-36	35,058	F	M*	M*	F
ST-40	41,720	M	Indet	Indet	M
ST-46	42,606	M	Indet	M*	M
**RTKT-23**	42,930	F*	F	F*	M
ST-51	45,661	M	M	M	M
ST-27A	47,008	F	F	F*	F
ST-47	56,495	M	F*	F*	M
ST-10	66,934	M	M	M	M
ST-5B	75,045	Indet	Indet	M	M
ST-57A	88,870	F	F	F*	F
**RTKT-20**	95,017	Indet	F	F	F
ST-15	131,763	Indet	M*	M	M
ST-44	136,755	M	M	M	M
ST-26	140,759	Indet	M*	M	M
ST-58A	174,112	F*	F	F*	F
**RTKT-7A**	186,704	F*	F	F*	F
ST-7	218,316	Indet	M	M	M
**RTKT-16**	298,184	Indet	F	F*	F
ST-32	311,636	Indet	M	M	M
ST-12	577,053	M*	M	M	M
ST-42	638,050	Indet	M	M	M
ST-9	737,923	Indet	M	M	M
**RTKT-10**	820,699	F*	F	F	F
**RTKT-17**	964,774	Indet	M	M	M
ST-52	1,052,930	M*	M	M	M
ST-56	1,404,346	M*	M	M	M
ST-6	1,581,177	F	F	F	F
ST-62	6,376,553	Indet	F	F	F
ST-63	10,796,470	Indet	M	M	M
ST-53	22,121,564	F	F	F	F
ST-54	24,679,707	Indet	F	F	F
ST-48	39,132,506	F	F	F*	F

The archaeological sites *Síi Túupentak* and *Rummey Ta Kuččuwiš Tiprectak* are abbreviated as ST and RTKT.

### Sex estimation as a function of DNA quality

**Figure 2:**
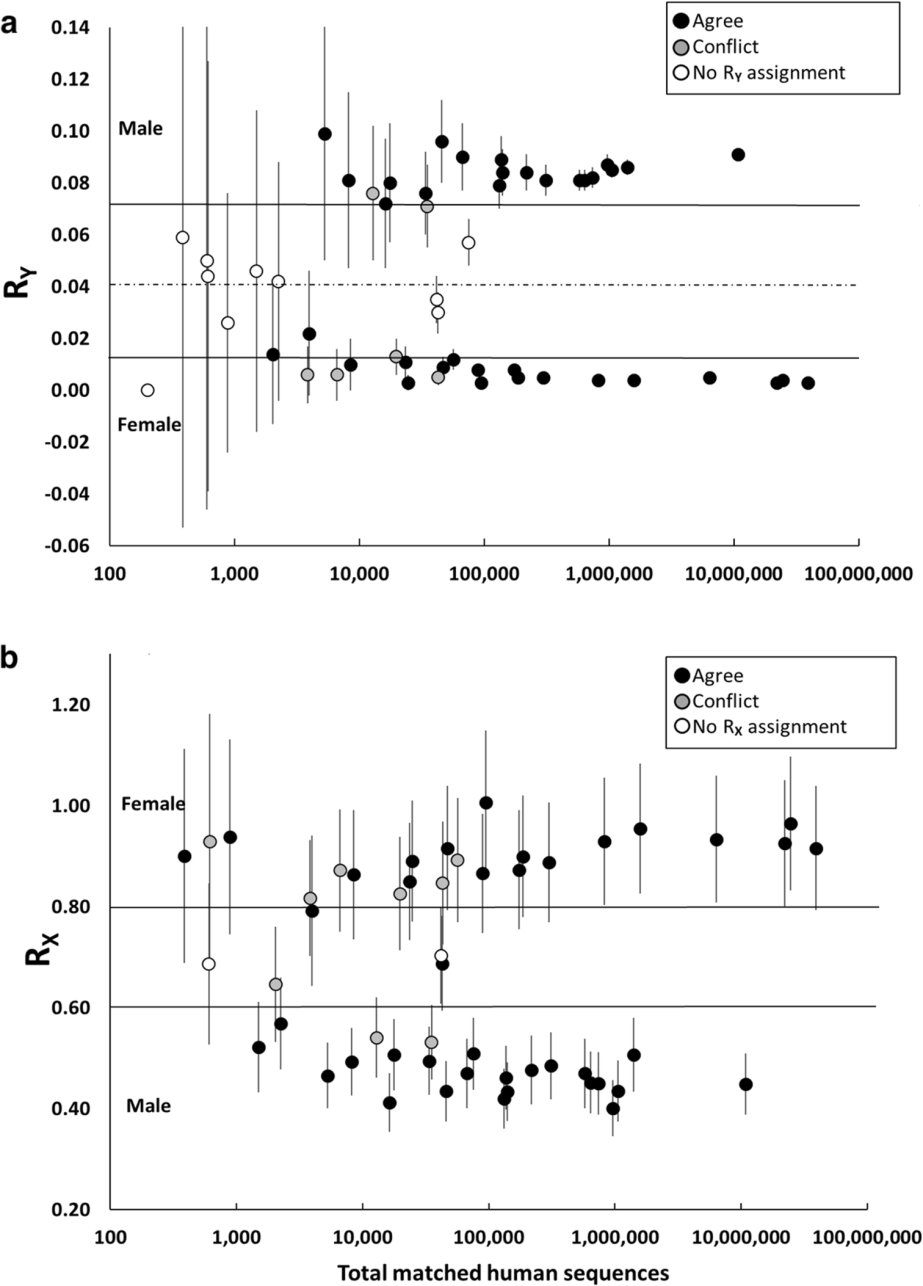
Consistency of sex estimation as a function of DNA data quality. Matching samples were processed for both proteomic and genomic sex estimation using the R_Y_ (**a**) and R_X_ (**b**) method^25,27^. In Figure 2a, genomic R_Y_ ratios with 95% confidence intervals (plotted as error bars) are shown as a function of DNA quality (total DNA read number) following Skoglund et al. 2013. Genomic conditional estimates (“consistent with XX, but not XY” or, “consistent with XY, but not XX”) have 95% confidence intervals that cross thresholds for definitive XX or XY karyotype assignment. These thresholds are indicated on the chart with solid horizontal lines (male > 0.075, and female < 0.016). Indeterminate samples fall entirely between the two thresholds. In Figure 2b, genomic R_X_ values are plotted in a similar manner though thresholds for males and females follow Mittnik et al. 2016^27^ (male < 0.60 and female > 0.80). Black fill indicates genomic assignments that were consistent with proteomics, gray fill indicates estimates that conflicted with proteomics, and white fill indicates samples where genomic sex estimation was indeterminate.

To evaluate the causes of inconsistent sex estimates, we plotted the R_Y_ and R_X_ values as a function of the number of matched human sequences following the original down-sampled test plot in Skoglund et al.^25^ and indicated consistent, inconsistent and indeterminate sex estimation (Fig. 2a and b, Figure S1 and S2). It is apparent that all conflicting and indeterminate sex estimates occur below the minimum of 100,000 sequence reads mapping to the human genome recommended in Skoglund et al.^25^.

Listing all sex estimates by increasing number of total matched sequences shows a similar pattern for both the R_Y_ and R_X_ methods (Table 2). Among the 55 common samples, the last conflict occurred just below 60,000 sequence reads, and last indeterminate estimate at 75,000 sequence reads. Table 2 also shows no definitive genomic estimates at or below 1,000 sequence reads using the R_X_ method. In this study, the lowest number of matched sequences to yield a definitive sex estimate using the Rx method was 5,256 (Table 2). It is further apparent that conflicts below the 100,000-threshold occurred primarily among conditional sex-estimates. In fact, conditional DNA-based sex-estimates with less than 100,000 total sequences agreed with proteomics only about half of the time using R_X_ (9 of 20, 45%,) and 38% (5 of 14) of the time using R_Y_, suggesting that under these conditions DNA-based estimates were close to random. The R_Y_ method also resulted in two conflicts among definitive estimates, both of which were below 100,000 reads.

The same is true for conflicts between osteological sex estimation and genomic sex estimates under 100,000 sequence reads. While the numbers were smaller due to a higher indeterminate rate, 4 out of 7 and 5 out of 11 conflicts were obtained with R_Y_ and R_X_ methods respectively. In this case, osteological sex estimation included both conditional and definitive assignments.

It is important to point out that less than half of the 55 common samples met the 100,000 read threshold. Including the samples that failed for DNA reconstruction, only 21 of 55 common samples (38.1%) exceed the 100,000 threshold. Though slightly higher, the proportion of samples exceeding 100,000 human genome sequence reads in the larger set of 99 skeletons sampled for DNA was also below 50% (42 of 99, 42.4%) (Table S1) indicating a representative sampling.

### Sex estimation as a function of proteomic data quality

**Figure 3:**
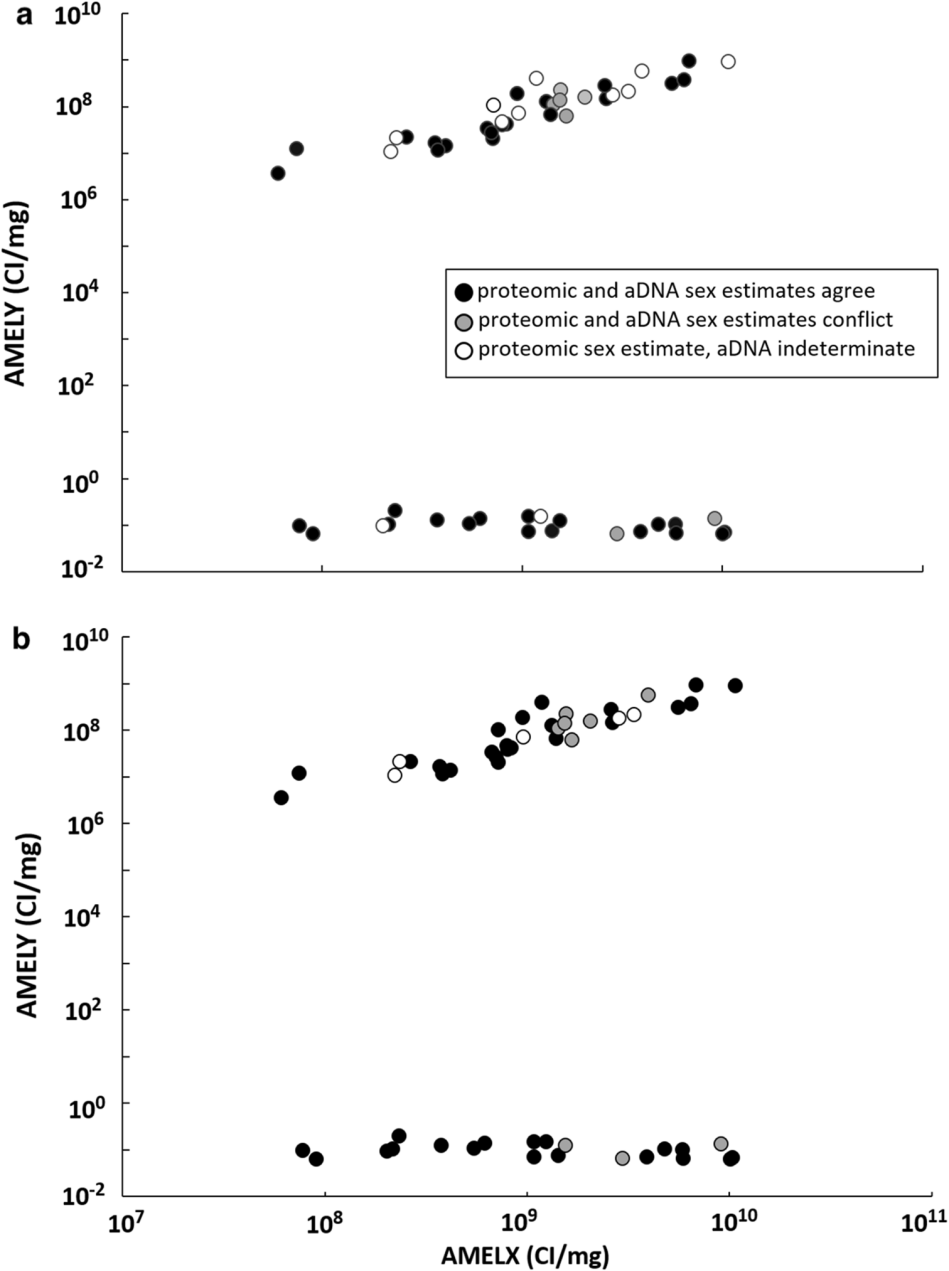
Consistency of sex estimation as a function of proteomic data quality. Matching samples were processed for both proteomic and genomic sex estimation using the R_Y_ (**a**) and R_X_ (**b**) method^25,27^. The cumulative ion intensity per mg enamel (CI/mg) for AMELY and AMELX peptides were plotted and consistency with both R_Y_ and R_X_ sex estimates indicated. Agreements with DNA-based R_Y_ estimates are indicated by black fill, conflicts are gray, and indeterminate genomic estimations are white.

To evaluate whether low proteomic signals contributed to inconsistencies in sex estimates we compared where the conflicts occurred for normalized combined intensities of amelogenin peptides (Table S3, Fig. 3a and b). These occurred mostly at mid- to high signal levels for AMELX and AMELY. Inconsistencies occurred between 1.43 × 10^9^ and 9.11 × 10^9^ CI/mg AMELX for R_Y_ estimates (Fig. 3b) and between 1.54 × 10^9^ and 9.11 × 10^9^ CI/mg AMELX for R_X_ (Fig. 3b). Indeterminate DNA-based estimates occurred across the range of proteomic amelogenin signals.

There were six conflicts where proteomic identifications were male and genomic estimates were female. With the R_X_ method, all of these conflicts occurred among conditional estimates, while three were conditional and two were definitive using the R_Y_ method (Tables 1 and 2). Of the six conflicts, the lowest proteomic signal male that conflicted with DNA was CA-ALA-565/H, Burial 35 (Table S3). This sample had 11 peptides that were unique to the AMELY gene product (Figure. S3). To place this in context, male samples in the total burial cohort ranged from a low of six specific AMELY peptides (CA-ALA-565/H, Burial 63A) to a high of 251 specific AMELY peptides (x̄ = 81, median = 37). All male assignments, by having multiple unique peptides, meet proteomic guidelines for publication of a detected protein and were considered definitive^63^. All replicate amelogenin analyses were within an order of magnitude (Table S4) and only a single AMELX peptide spectrum was detected in a blank run (Table S5). No specific AMELY peptides were detected in any blanks.

Overall, there were three conflicts where proteomic sex estimation was female and genomic was male (CA-ALA-704/H Burial 7C, CA-ALA-565/H Burials 29 and 36). In each of these cases the genomic assignments were conditionally male, while proteomics detected no AMELY peptides and had abundant AMELX peptides with relatively strong combined intensities corresponding to Pr(F) values of 0.95, 0.97 and 0.99 respectively (Table S3). The lowest AMELX signal sample (CA-ALA-565/H, Burial 62) had a proteomic female sex estimation with a Pr(F) value of 0.68. This was a repeat of an earlier sample that resulted in lower amelogenin yields and a Pr(F) value of 0.29, an indeterminate proteomic sex estimate. However, while this was the lowest proteomic signal it was supported by a definitive genomic female sex estimation with high quality DNA (total reads = 6.4 × 10^6^). Data for all duplicate proteomic samples are listed in Table S4.

### Relative preservation of amelogenin peptide and DNA signal quality

**Figure 4:**
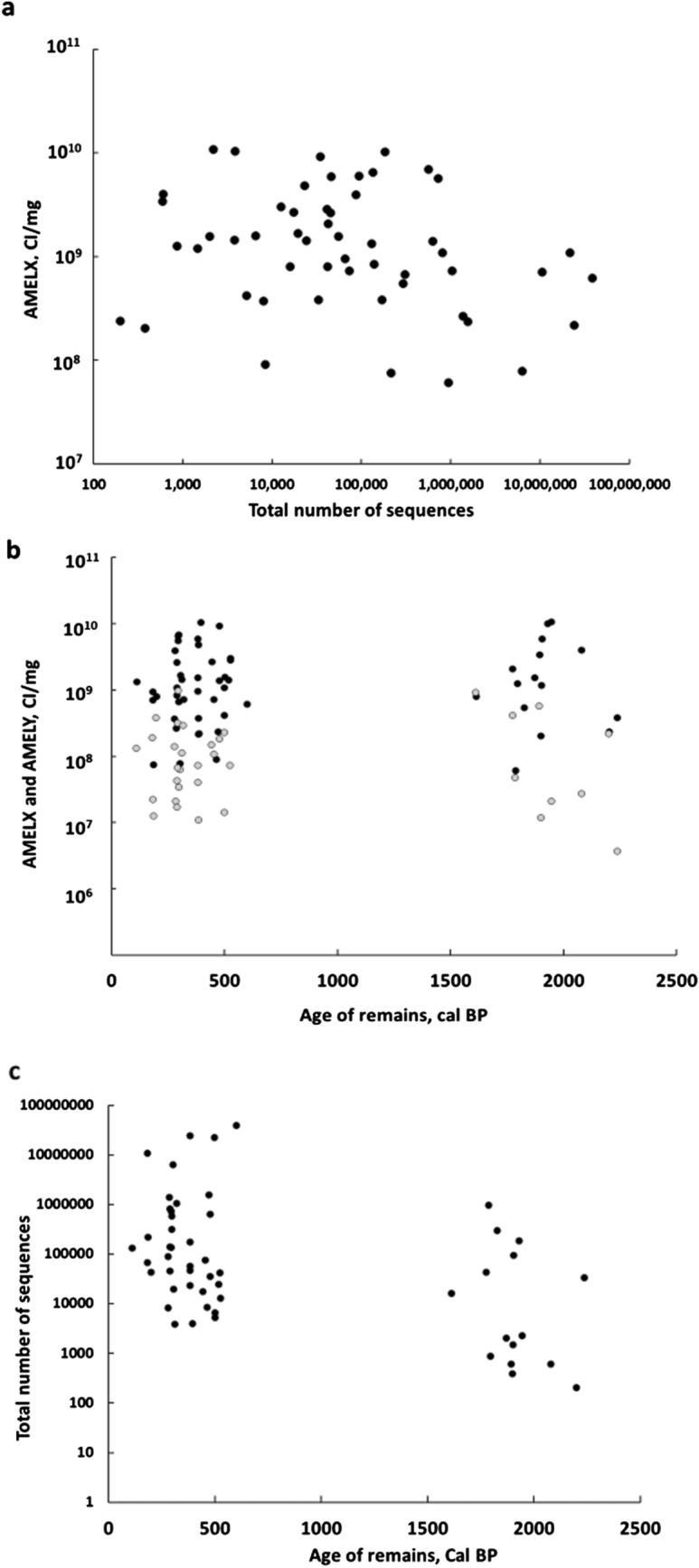
Correlation of Amelogenin and DNA signal intensity. (**a**) Intensity of AMELX_HUMAN signal (CI/mg) as a function of the total number of matched human DNA sequence reads. (**b**) Intensity of AMELX_HUMAN signal (CI/mg) as a function of the age of skeletal remains (cal BP), (**c**) The total number of matched human DNA sequences as a function of the age of skeletal remains (cal BP).

Since the efficacy of sex estimation is dependent on the quality of DNA and peptide signals, we compared matching signal types taken from each skeleton. Degradation of DNA and protein, particularly from the same sample, would be predicted to affect both signals and result in a positive relationship. Matching AMELX (CI/mg) was plotted as a function of matching total DNA reads (Fig. 4a). In order to accommodate the large range of signal, and allow variation to approximate a normal distribution, all values were transformed logarithmically prior to linear regression^64^. No significant linear relationship between the variables was detected in spite of the high power of the sample (df = 51, *p* = 0.09). This result is consistent with other studies, although sampling from different skeletal locations may have introduced variation^42,65^. Results of all statistical analyses can be found in supplemental materials.

Another approach is to compare the values of each variable as a function of archaeological age. With one exception (a more recent sample from CA-ALA-704/H), samples from the two sites fit into two discrete age categories. Late/Historic Period samples from CA-ALA-565/H span 600–100 cal BP, while EMT/Middle 1 Period samples from CA-ALA-704/H date between 2,240–1,610 cal BP (Fig. 4b). The range of signal for amelogenin peptides, which were transformed logarithmically using a base of 10, averaged 9.03 ± 0.56 orders of magnitude for the Late/Historic Period samples and 9.08 ± 0.63 for EMT/Middle Period samples. An independent t-test found no significant difference in AMELX signal between the two groups (two-tailed, df = 53, *p* = 0.78). This supports a stable proteomic signal over this timeframe (roughly 2,000 years) and is consistent with previously published observations^33^.

The same was not the case for DNA quality. The range of logarithmically transformed total DNA reads averaged 5.13 ± 1.10 orders of magnitude for Late/Historic Period samples and 4.05 ± 1.25 orders of magnitude for EMT/Middle 1 Period samples, a reduction of about an order of magnitude in the older samples (Fig. 4c). An independent t-test found the difference between these two groups to be significant (2-tailed, df = 51, *p* = 0.002, Supplemental Material). These results support a working hypothesis of independent or orthogonal signals for ancient DNA and amelogenin protein. The practical result is that low signal DNA samples may have high amelogenin signals and vice versa, and that combining information from both DNA and proteomic methods will mutually support concurring estimates and correct for conflicting conditional estimates.

These data confirm the implications from the analysis of conflicting sex estimations described above. Conflicting sex estimates started to become evident in samples with poorer quality DNA, below the threshold of 100,000 reads (Fig. 2). No such pattern was clear when mapping conflicting sex estimates onto proteomic data. Conflicting sex estimates occurred across different proteomic data quality levels (Fig. 3). This is supported by finding that proteomic data quality is orthogonal to DNA data quality (Fig. 4A). Together these imply that conflicting sex estimates are due to poor quality DNA and not proteomic data. This is supported by the finding that proteomic data quality is more stable compared to DNA (Fig. 4b and c).

## Discussion

To the best of our knowledge, this is the largest archaeological study to compare different molecular and osteological methods of sex estimation. Because analyses of shotgun-sequenced DNA (using both R_Y_ and R_X_ methods), amelogenin protein, and osteological markers were made on the same set of individuals, matching datasets allowed us to make direct comparisons of the performance of the three techniques and develop a framework for managing inevitable conflicting sex estimates. When low values or confidence scores are obtained for any one method, the result can be compared to other methods and help determine the thresholds at which inconsistent sex estimation begin to occur.

Proteomics was the most sensitive method (i.e., provided estimates for the highest percentage of samples where all methods were applied), followed by genomic-based sex estimates, and osteology. Overall, there was a high amount of consistency between the different methods. We observed total agreement between the three methods where osteology had definitive sex estimates, when DNA had more than 100,000 total reads, and when R_X_ values resulted in definitive sex estimates.

Osteology offers a highly reliable, relatively fast means of estimating sex, although extensive training is required. Osteological methods are especially valuable as there are many contexts where molecular techniques cannot be applied due to cost and preference of the descendent community. However, as shown here, the osteological method is limited to adult skeletons with preserved sexually dimorphic markers, such as *os coxae* and crania. Nonetheless, it is highly reliable when preservation is good. All definitive osteological sex estimates concurred with definitive DNA and proteomic estimates. There were only four and five discrepancies, respectively, with conditional R_Y_- and R_X_-based sex estimates, and just one of a total of twelve conditional osteological sex estimates disagreed with a proteomic sex estimate.

A strong benefit of high-throughput shotgun-sequenced DNA-based assignment of sex is that it can piggyback off of analyses performed for other reasons, such as information on the ancestry of an individual or evidence of disease^21,26,66–69^. It can also be applied to a variety of human tissues, including bone, skin, hair, and teeth. This provides more flexibility than the analysis of amelogenin protein, which is restricted to tooth enamel.

Of the two DNA-based methods, the R_X_ ratio was more sensitive than the ratio based on sex chromosome reads (R_Y_) (Table 1), with more samples resulting in a sex estimate, although many of these additional estimates were conditional. For both of the DNA-based methods, conditional estimates had a high rate of inconsistency with proteomic and osteological sex estimates (Tables 1 and 2). Definitive R_X_ estimates were uniformly consistent with osteological and proteomic sex estimates, while definitive R_Y_ estimates produced two conflicts with proteomic sex estimation. Both of these conflicts occurred below 100,000 DNA sequence reads.

In this study, the limits of 100,000 total reads originally proposed in Skoglund et al.^25^ were supported by proteomic sex estimates and osteological sex estimates. All conflicts with proteomic and osteological sex estimates occurred below this threshold. Thus, caution should be applied to genomic sex estimates when the total number of mapped human sequences is below 100,000. This is particularly so for conditional, or ‘consistent with. . .’, estimates, whether they are made using R_Y_ or R_X_ criteria. While no definitive R_X_ sex estimates conflicted with proteomics or osteology, and no conditional estimates above 100,000 total reads conflicted, conditional estimates made on samples below 100,000 reads agreed with proteomics at a rate only slightly better than chance alone (5/14 for R_Y_ and 9/20 for R_X_). While the numbers were smaller the same phenomenon was observed for conflicts with osteological sex estimation.

Given that no definitive R_X_ sex estimates conflicted with proteomics or osteology, it may be possible to increase the total number of confident genomic sex estimates by combining definitive R_X_ estimates below 100,000 reads with R_Y_ or R_X_ estimates (definitive and conditional) that have more than 100,000 total matched human sequences. This would increase the number of confident genomic sex estimates by 16.4% (from 21 to 30 individuals). At what point definitive R_X_ estimates become less reliable, however, remains an open question. The much lower threshold of 1,000 reads originally proposed for the R_X_ method^27^ could not be confirmed here as no definitive R_X_ sex estimates were obtained below about 5,000 reads.

Refinements in analysis of shotgun sequenced DNA could also increase confident genomic sex estimates. Researchers may conduct a detailed analysis of sex chromosome sequences to exclude homologous regions and provide a higher confidence of sex chromosome assignment. The use of targeted SNP data to conduct sex estimation helps in this regard. The resulting ratios of average sex chromosome to autosomal coverage based on X and Y rates may reduce chromosome miss-assignment and increase the signal separation between males and females^66,68,70^. The use of SNP rates and affirmation or otherwise with proteomic sex estimation is the focus of additional study.

In contrast to other methods, sex estimation based on amelogenin proteins was more sensitive, with assignments made on all samples, including those that failed for DNA sex estimation and samples from two cremated individuals (Table S1). All proteomic male sex estimates were based on confident assignments of multiple AMELY peptides and were considered determinate^62^. Female assignments were more complex, but the calculated probabilities of female sex (Pr(F)) were generally high and lower probabilities were corroborated with high quality DNA data^33^.

Proteomic sex estimation exploits the fact that the highly characterized sex-chromosome-specific amelogenin gene family is expressed as proteins in the most robust tissue in the human body, enamel^33,34,37,58,71^. The proteins are cleaved into peptides in situ, as part of enamel formation during tooth biogenesis^50,72,73^. In order to extract and analyze this peptide population, researchers need to demineralize the enamel and most use acid-based^33–36,45,46,55,58,74^ approaches. There are two analytical options: a targeted approach focused on a limited number of specific amelogenin peptides^34,36,54^, or a shotgun proteomics approach that seeks to identify all proteins in the proteome and then selectively measure all amelogenin peptides bioinformatically after peptide spectral matching^35,45,46,54,55^. This study takes the later approach^33^. By comprehensively identifying and measuring all unambiguous AMELX and AMELY peptides, the chromosome-specific signal is maximized. Stochastic effects that may result from any one peptide will be minimized. The approach is validated in this study by the high sensitivity of proteomic sex estimation, the stability of proteomic data over time, and the finding that there was no functional correlation between proteomic and genomic signals.

Because the amelogenin peptide signal appears to be independent from DNA-based sex estimation, confident proteomic sex estimates can occur in samples with low or absent levels of DNA, and vice versa. In this study, amelogenin peptide signal remained stable over approximately 2,000 years while DNA levels significantly decreased in the older samples (Fig. 4b and c). Stability of the proteomic signal may be a function of competing factors. Amelogenin peptides adhere to the biomineral interface or are incorporated into the apatite matrix, reducing peptide flexibility and reactivity^33,75^. Over time, proteins that are less incorporated in the mineral matrix, such as extracellular matrix proteins, will degrade at a faster rate resulting in a less complex proteome that is relatively enriched with amelogenin peptides^33,35^. As a result, remaining amelogenin peptides are more likely to be targeted by the mass spectrometry instrument for fragmentation, increasing the cumulative signal.

The utility and complementarity of proteomic, genomic, and osteological techniques was related to differences in mortuary treatments and preservation encountered in this study. Proteomics was able to estimate sex in several cases where genomics failed, including skeletal remains from one cremation (CA-ALA-565/H, Burial 30). On the other hand, not all burials contained teeth with sufficient enamel, which precluded analysis of amelogenin protein. This was particularly true for cremated remains at CA-ALA-565/H, which were secondarily interred and formed a sizeable portion of the burial population (21%). Overall, it was possible for genomic sex estimation to be attempted on a larger number of burials, even though proteomics had greater sensitivity. Combining proteomic, genomic, and osteological data produced highly comprehensive and confident sex estimations for the burial populations analyzed in this study. This allowed detailed male and female survival functions to be constructed, which enabled us to better detect sex-biased mortality patterns among the subadult population at CA-ALA-565/H. These sex-biased mortality patterns are the subject of a forthcoming paper. Future systematic comparisons are needed to understand the relative strengths of these molecular techniques with respect to various mortuary treatments and over a wider range of environmental and temporal contexts.

Finally, and most importantly for the Muwekma Ohlone Tribe of the San Francisco Bay Area, accurate sex determination provides a greater perspective on the persona of each individual, rather than the nebulous "indeterminate" status of a person or child. Tribal members and representatives of the scientific community are collectively looking into the lives and tragedy of the death of people from the past. If it was not for their sacrifice, struggles, and commitment to their families, Muwekma Ohlone would not survive to this day. Today, the Muwekma Ohlone celebrate the lives of their ancestors by retelling some of their history and stories through archaeology, and ultimately honor them when they are returned to the *warep* (roughly translated as “the earth”), where their loved ones originally placed them with love and respect.

## Conclusions

A large-scale comparison of proteomic, genomic, and osteological methods of sex estimation provides a unique opportunity for contrasting the benefits and limits of each technique. We empirically demonstrate that the thresholds of 100,000 total and 3,000 sex chromosome reads for genomic sex estimation is impactful; all conflicts occur below this threshold and no inconsistencies occur above it. In particular, conditional “consistent with . . .” estimates below this threshold were effectively random with respect to proteomic and osteological determinations.

The study showed that osteological sex estimation is reliable (i.e., consistent with other techniques when sample signal is high), but has a high rate of indeterminate sex assignments when fragmentary and juvenile remains are assessed. Genomic methods help to extend sex estimation to many juvenile or fragmentary remains, but had a high rate of conflict with osteology or proteomic estimates for conditional sex assignments below the 100,000 total mapped read threshold. In the event of a conflict in sex estimation, these conditional DNA-based estimates should be disregarded in favor of other methods. Proteomic sex estimation was the most sensitive technique, providing results in all remains tested, due in part to the stability of the amelogenin peptide signal, but was contingent upon the preservation of dentition associated with each burial. Conflicts between proteomic and DNA based estimates could be attributed to the different level of stability and signal variation between the two types of biomolecules. To obtain the greatest coverage and confidence in sex estimates among archaeological burial populations, proteomic approaches should be combined with osteological and genomic methods.

## Methods

### Osteology

To estimate osteological sex, 20 unique traits were observed for each individual when present in a laboratory setting, and scored to indicate a prevalence of male or female for each trait (Table S6). These 20 traits included nine that were observed on the *os coxae* (subpubic concavity, shape of pubis, ventral arc, doral pits, acetabulum size, greater sciatic notch, preauricular sulcus, auricular surface, and acetabulum dimensions), six on the cranium and mandible (nuchal crest, mastoid process, supraorbital margin, supraorbital ridge, mental eminence, and ascending ramus), and five that were quantitatively categorized for robusticity (glenoid fossa size, vertical diameter of humeral head, maximum width of humeral epicondyle, maximum diameter of femoral head, and maximum width of femoral bicondyle). All assessed traits have previously been shown to contribute to accurate sex estimation^16,76,77^. Due to the complexity of human sexual dimorphism, the scores for these 20 traits were then comprehensively evaluated relative to the local population to best determine the sex of the individuals (Table S6). In infants and children who died before puberty, current standard sexually dimorphic skeletal traits had not yet developed and could not be scored in this study.

### Genomics

Whole genomic DNA extraction was conducted on a total of 99 ancient tooth and bone samples (71 individuals from CA-ALA-565/H, including seven samples that failed for reconstruction, and 28 individuals from CA-ALA-704/H; Table S2) following methods described in Cui et al.^78^. All genomic libraries exhibited expected DNA damage supporting the authentication of the DNA results. All ancient DNA laboratory work was conducted in a laboratory that is dedicated exclusively to studies involving ancient DNA at the Carl R. Woese Institute for Genomic Biology, University of Illinois at Urbana-Champaign (UIUC). All DNA extraction and genomic library preparation rounds included negative controls to account for DNA contamination. Libraries were constructed using the NEBNext Ultra II DNA Library Prep kit and NEBNext Multiplex Oligos (Unique Dual Indexes) for Illumina, and shotgun-sequenced on a HiSeq 4,000 platform at the UIUC Core Sequencing Facility.

Samples were de-multiplexed and trimmed to have a minimum sequence length of 25 bp using the program FastP v. 0.19.6^79^, and DNA sequence reads were aligned to the human *hg19* reference genome (GRCh37 – Genbank accession number: GCA_000001405) using Burrows-Wheeler alignment in BWA v. 0. 7.15^80^. Aligned sequences were transformed to BAM format in SAMtools v. 1.1^81^ and filtered to remove unmapped reads and reads with a quality score less than 30. PCR duplicates were marked and removed with the Picard Toolkit v. 2.10.1 (“Picard Toolkit” 2019, Broad Institute), and index statistics for BAM files were generated using “idxstats” in SAMtools^81^. R_Y_ and R_X_ ratios were calculated following methods described in Skoglund et al.^25^ and Mittnik et al.^27^. Mapdamage 2.0 was used to check for DNA damage associated with ancient DNA^49^.

### Proteomics

Amelogenin peptides were extracted and analyzed from the tooth enamel of 55 individuals (39 individuals from *Síi Túupentak* and 16 individuals from *Rummey Ta Kuččuwiš Tiprectak*; Table 2, S1 and S2). All surfaces and tools were thoroughly cleaned between samples and sample blanks were prepared with each batch. Washing runs with saw-tooth gradients on liquid chromatography were employed between each sample and periodic blank runs were used to monitor sample carryover. Proteomic methods followed those described in Parker et al.^24^ with the following changes. Mass spectrometry datasets (.RAW format) were processed with PEAKS (10.0) peptide matching software (Bioinformatics Solutions Inc., Waterloo, ON). Error tolerance for matching peptide spectral assignment was set to 10 ppm for precursor mass and 0.04 Da for fragment ions. AMELX_HUMAN signals (CI/mg) were log transformed and then solved for Pr(F) using the equation Pr(F) = 1.0 + (0.059–1.0)/(1 + (x/7.54)^13.99^ where “x” is the logarithm (base 10) of the AMELX_HUMAN^24^. Samples with a Pr(F) < 0.5 were considered indeterminate for proteomic sex estimation. Full details of the proteomic methods are provided in supplemental information^82–84^.

## Data availability

The mass spectrometry proteomics data, along with customized protein reference library, have been deposited to the ProteomeXchange Consortium via the PRIDE partner repository with the accession number PXD016076 (https://www.proteomexchange.org^85^.

## Supplementary information

Supplementary file
