# Supplementary material for: A comparison of proteomic, genomic, and osteological methods of archaeological sex estimation
Source: Sci Rep. 2020 Jul 17;10:11897. doi: 10.1038/s41598-020-68550-w (PMC7368048; doi:10.1038/s41598-020-68550-w)

**SUPPLEMENTAL MATERIALS**

**Manuscript Title: A Comparison of Proteomic, Genomic, and Osteological Methods of Archaeological Sex Estimation**

**Authors:**

Tammy Buonasera^1,2^*, Jelmer Eerkens^2^, Alida de Flamingh^3^, Laurel Engbring^4^, Julia Yip^1^, Hongjie Li^5^, Randall Haas^2^, Diane DiGiuseppe^6^, Dave Grant^6^, Michelle Salemi^7^, Charlene Nijmeh^8^, Monica Arellano^8^, Alan Leventhal^8,9^, Brett Phinney^7^, Brian F. Byrd^4^, Ripan S. Malhi^3,5,10^, Glendon Parker^1^*

**Author Affiliations:**

1. Department of Environmental Toxicology, University of California, Davis

2. Department of Anthropology, University of California, Davis

3. Program in Ecology, Evolution and Conservation Biology, University of Illinois at Urbana-

Champaign

4. Far Western Anthropological Research Group, Inc., Davis, California

5. Department of Anthropology, University of Illinois at Urbana-Champaign

6. D&D Osteological Services, LLC, San Jose, California

7. Proteomic Core Facility, Genome Center, University of California, Davis, California

8. Muwekma Ohlone Tribe of the San Francisco Bay Area, Milpitas, California

9. Department of Anthropology, San Jose State University, San Jose, California

10. Carl R. Woese Institute for Genomic Biology, University of Illinois at Urbana-Champaign

***Corresponding Authors**

Rm 5241B Meyer Hall,

1 Shields Ave,

Davis, CA 95616 USA

p) 530) 752-9870

e) [gjparker@ucdavis.edu](mailto:gjparker@ucdavis.edu);

[tybuonasera@ucdavis.edu](mailto:tybuonasera@ucdavis.edu)


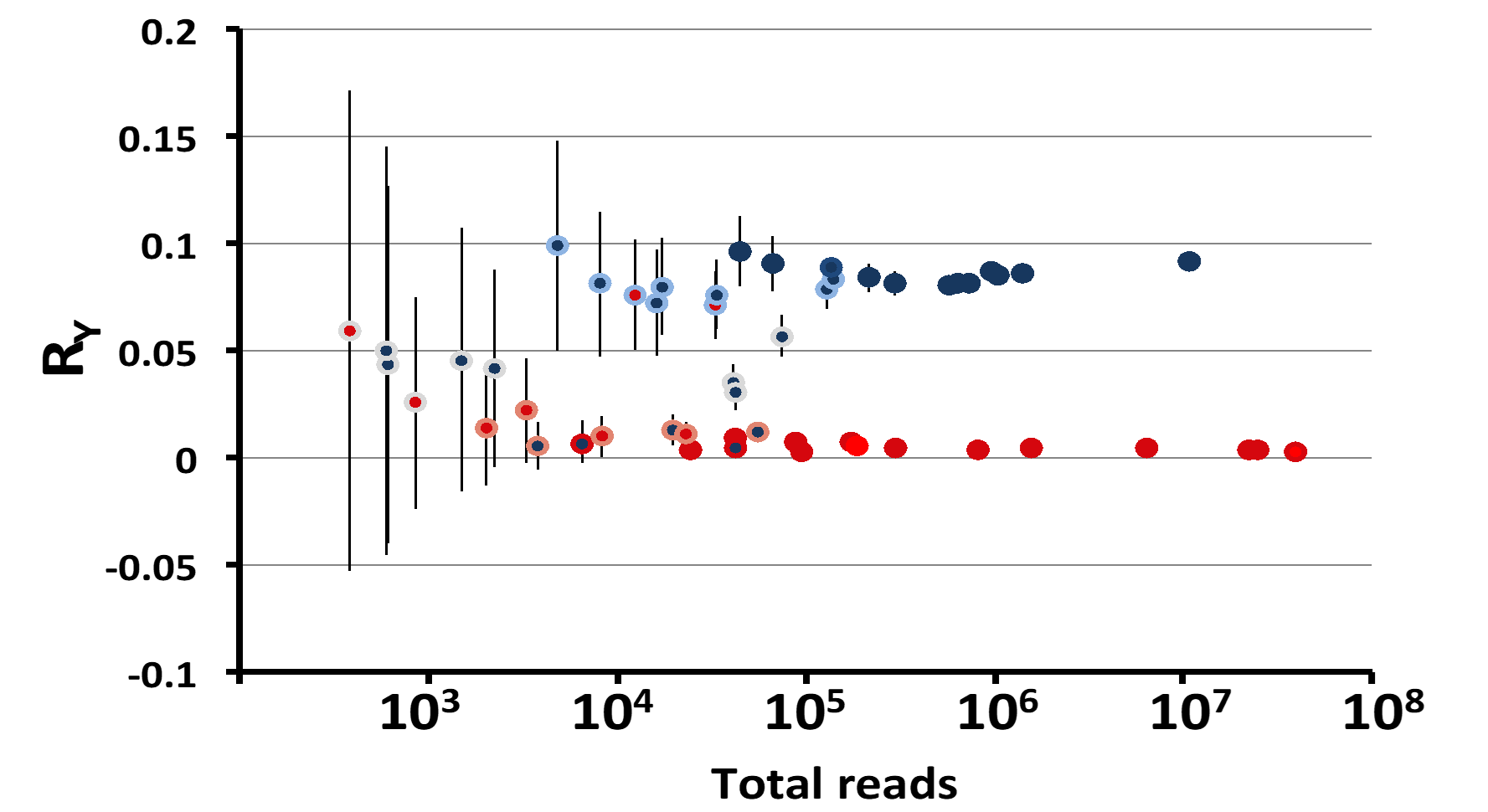


**Figure S1.** The level of AMELY_HUMAN and AMELX_HUMAN (Cumulative Ion Intensity per mg enamel, CI/mg) was used to proteomically estimate the sex of each individual (inner circle: red = female; blue = male) and the DNA reads from each sex chromosome was used to estimate the sex of each individual with the R_Y_ method (outer circle: red = female; light red = consistent with female, grey = not assigned, light blue = consistent with male, blue = male). Datapoints are plotted according to the scheme introduced by Skoglund et al.(Skoglund, et al., 2013).


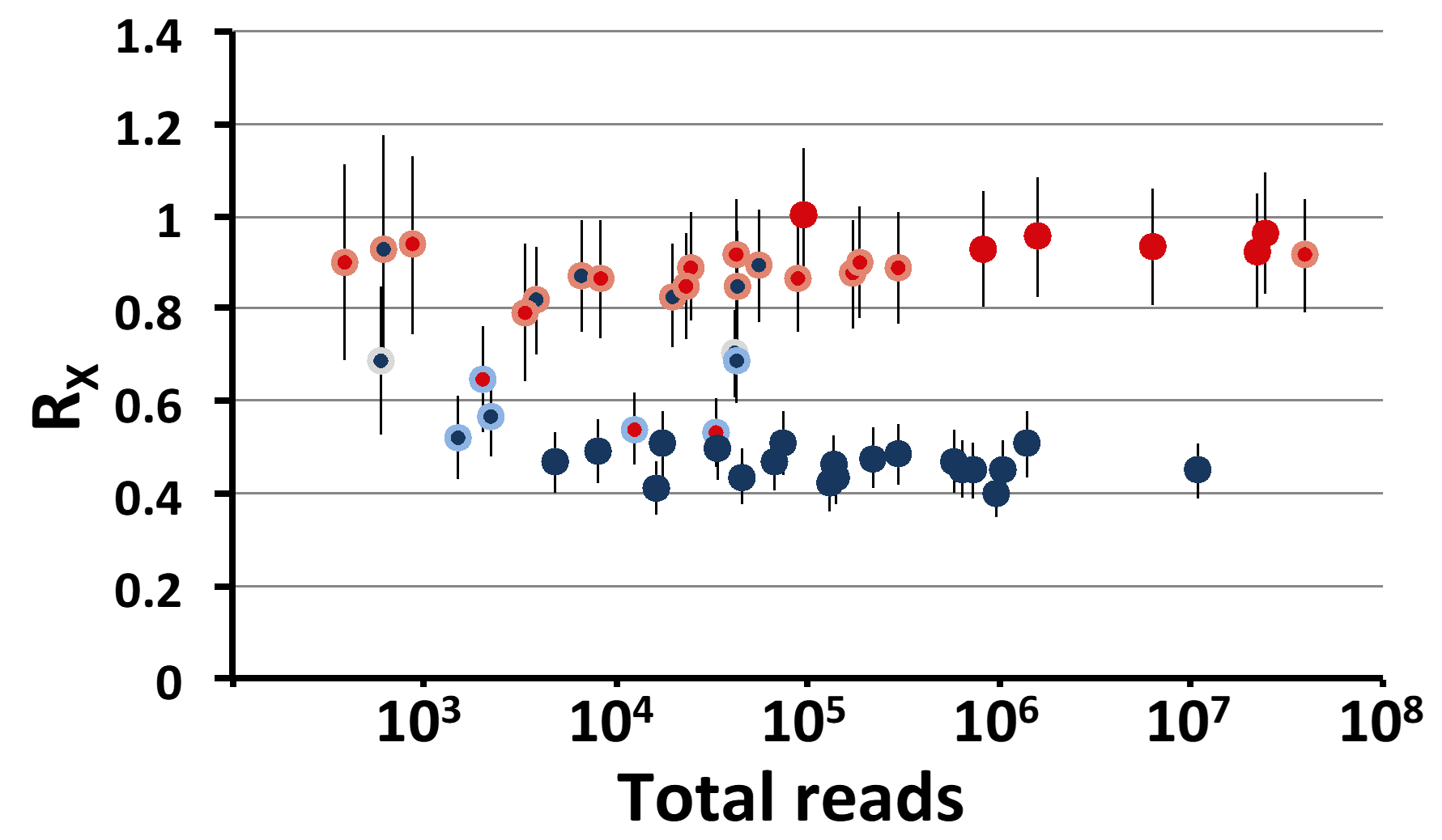


**Figure S2.** The level of AMELY_HUMAN and AMELX_HUMAN (Cumulative Ion Intensity per mg enamel, CI/mg) was used to proteomically estimate the sex of each individual (inner circle: red = female; blue = male) and the DNA reads from each sex chromosome was used to estimate the sex of each individual with the R_X_ method (outer circle: red = female; light red = consistent with female, grey = not assigned, light blue = consistent with male, blue = male). Datapoints are plotted according to the scheme introduced by Skoglund et al.(Skoglund, et al., 2013) using the criteria developed by Mittnik et al.(Mittnik, et al., 2016).


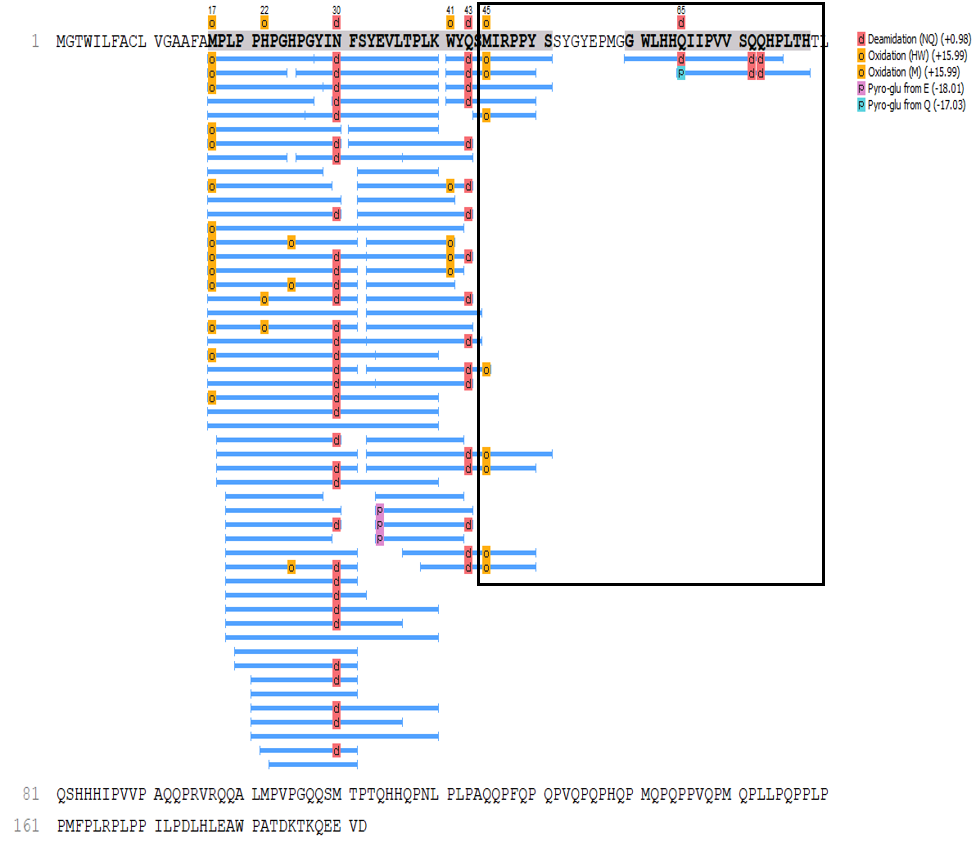


**Figure S3.** AMELY isoform 1 (Uniprot accession number: Q99218-1) peptide coverage for B35 (*Síi Túupentak*) using the PEAKS Peptide Spectra Matching algorithm(Zhang, et al., 2012). Peptide sequences detected in this sample are shown in blue. Amino acid sequences specific to AMELY are indicated by the black outline with all peptides within this range containing amino acids residues specific for the AMELY isoform 1 gene product.


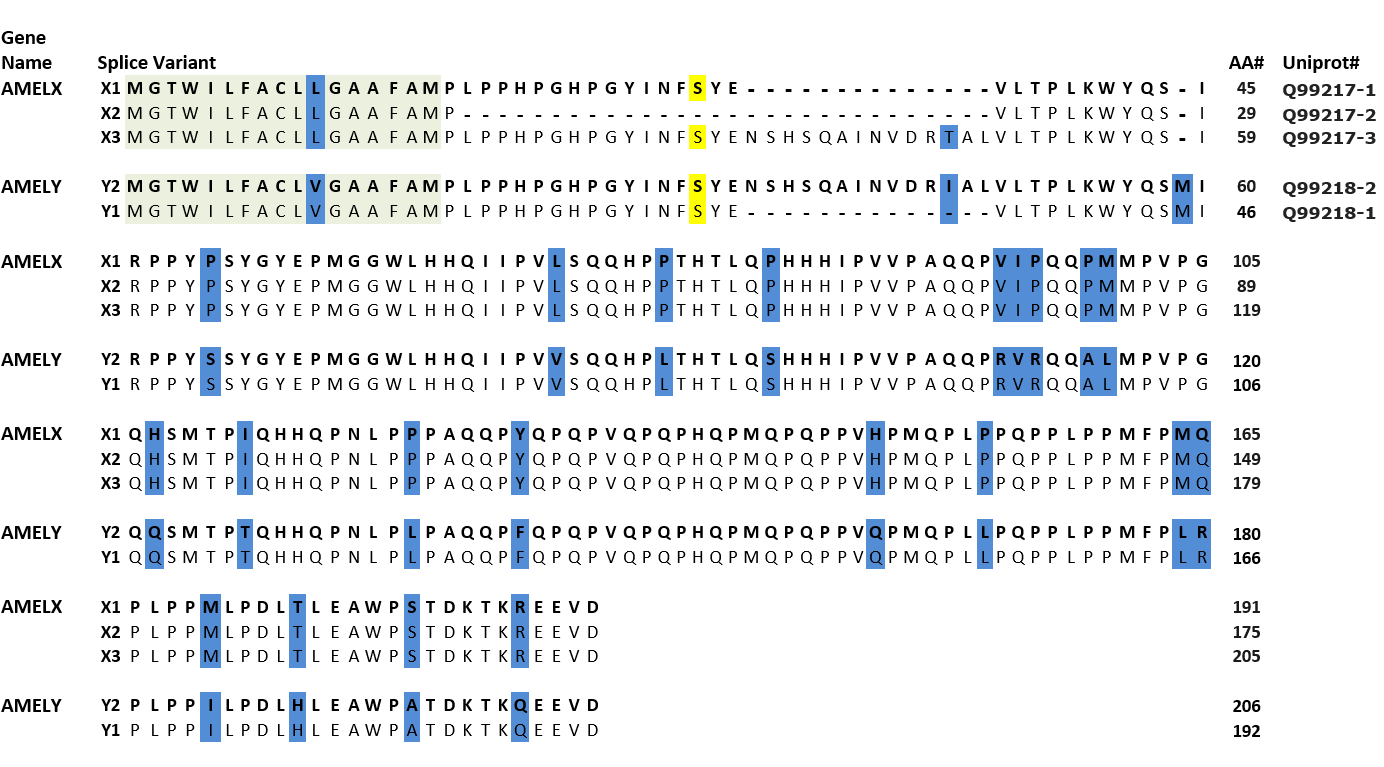


**Figure S4.** Amino Acid Variation in Amelogenin Sex-Specific Isoforms. Amelogenin genes are transcribed in three AMELX isoforms and two AMELY isoforms (Salido, et al., 1992, Simmer, 1995). Amino acid variation between the two proteins are indicated in blue squares. The putative signal peptide is indicated in green, the documented site of phosphorylation (S15) is indicated in yellow(Connelly, et al., 2016). UNIPROT protein accession numbers are indicated.

References Cited

Connelly, C., Cicuto, T., Leavitt, J., Petty, A., Litman, A., Margolis, H.C., Gerdon, A.E., 2016. Dynamic interactions of amelogenin with hydroxyapatite surfaces are dependent on protein phosphorylation and solution pH, *Colloids Surf B Biointerfaces* **148**, 377-384. 10.1016/j.colsurfb.2016.09.010.

Deutsch, E.W., Csordas, A., Sun, Z., Jarnuczak, A., Perez-Riverol, Y., Ternent, T., Campbell, D.S., Bernal-Llinares, M., Okuda, S., Kawano, S., Moritz, R.L., Carver, J.J., Wang, M., Ishihama, Y., Bandeira, N., Hermjakob, H., Vizcaino, J.A., 2017. The ProteomeXchange consortium in 2017: supporting the cultural change in proteomics public data deposition, *Nucleic Acids Res* **45**, D1100-D1106. 10.1093/nar/gkw936.

Mittnik, A., Wang, C.-C., Svoboda, J., Krause, J., 2016. A Molecular Approach to the Sexing of the Triple Burial at the Upper Paleolithic Site of Dolní Věstonice, *PLoS ONE* **11**, e0163019. 10.1371/journal.pone.0163019.

Salido, E.C., Yen, P.H., Koprivnikar, K., Yu, L.C., Shapiro, L.J., 1992. The human enamel protein gene amelogenin is expressed from both the X and the Y chromosomes, *Am J Hum Genet* **50**, 303-316.

Simmer, J.P., 1995. Alternative splicing of amelogenins, *Connect Tissue Res* **32**, 131-136.

Skoglund, P., Storå, J., Götherström, A., Jakobsson, M., 2013. Accurate sex identification of ancient human remains using DNA shotgun sequencing, *Journal of Archaeological Science* **40**, 4477-4482. <http://dx.doi.org/10.1016/j.jas.2013.07.004>.

Zhang, J., Xin, L., Shan, B., Chen, W., Xie, M., Yuen, D., Zhang, W., Zhang, Z., Lajoie, G.A., Ma, B., 2012. PEAKS DB: de novo sequencing assisted database search for sensitive and accurate peptide identification, *Mol Cell Proteomics* **11**, M111 010587. 10.1074/mcp.M111.010587.

**Supplemental Tables**

Table S1. Data for all human burials excavated at the archaeological sites *Síi Túupentak* (CA-ALA-565/H) and *Rummey Ta Kuččuwiš Tiprectak (*CA-ALA-704/H).

Radiocarbon dates are given as the median intercept of calibrated radiocarbon years BP. Osteological data include approximate completeness of the skeletal remains (% complete), age categories: Fetus (< 0 years), Infant (0-3 years), Child (4-10 years), Juvenile (11-17 years), Young Adult (18-34 years), Middle Adult (35-49 years), Older Adult (> 50 years), Adult (age uncertain), and sex estimates. Conditional sex estimates are designated as probable (Prob.) or possible (Poss.) with the former having greater certainty. Cases where osteological sex estimates could not be made are indeterminate (Indet). Genomic data include numbers of matched human DNA sequences (Total Reads), along with Ry and Rx values with 95% confidence intervals (RY ± range, and RX ± range). Genomic sex estimates are definitively female or male (XX or XY), conditionally female or male (consistant with XX, but not XY; or consistant with XY, but not XX). Several cases are not assigned due to calculated values (Not Assigned) or because DNA could not be reconstructed (Failed for reconst.). Proteomics data include the total cumulative ion signal intensity for AMELX_HUMAN and AMELY_HUMAN peptides (AMELX (CI) and AMELY (CI), as well as the probability of a true female assignment.

Table S2. Osteological, genomic, and proteomic sex estimates for 55 human burials from archaeological sites *Síi Túupentak* (CA-ALA-565/H) and *Rummey Ta Kuččuwiš Tiprectak (*CA-ALA-704/H)*.*

Radiocarbon dates are given as the median intercept of calibrated radiocarbon years BP. Osteological data include approximate completeness of the skeletal remains (% complete), age categories: F (fetus, < 0 years), I (infant, 0-3 years), C (child, 4-10 years), J (juvenile, 11-17 years), YA (young adult, 18-34 years), MA (middle adult, 35-49 years), OA (older adult, > 50 years), A (adult, age uncertain), and sex estimates. Conditional sex estimates are designated as probable (Prob.) or possible (Poss.) with the former having greater certainty. Cases where osteological sex estimates could not be made are indeterminate (Indet). Genomic data include numbers of matched human DNA sequences (Total Reads), along with Ry and Rx values with 95% confidence intervals (RY ± range and RX ± range). Sex estimates are designated as definitively female or male (F or M), as conditionally female or male (F*or M*), or indeterminate (Indet). Proteomics data include the total cumulative ion signal intensity for AMELX_HUMAN and AMELY_HUMAN peptides (AMELX (CI) and AMELY (CI)), as well as the probability of a true female assignment.

**Table S6. Scoring of 20 unique traits for osteological estimation of sex.**

**Proteomic Laboratory Methods**

A small amount of enamel, ~20 mg, was removed from teeth by cutting with a clean double sided, diamond-coated steel disc (Brasseler Inc., Savannah, GA, HP medium), or by grinding with an unused carbide steel burr (Brasseler Inc, Savannah, GA, US# 7 SH round). Blades were cleaned with ultrasonication and triple rinsed with 95% ethanol between samples. Powdered or cut enamel samples (~ 20 mg) were demineralized by adding 200μL of 1.2M hydrochloric acid to 2 mL sample vial with seven 2.8 mM ceramic beads (Omni-International Inc.). Samples were milled for 3 minutes at 7000 rpm in a MagnaLyzer (Roche Inc.), then centrifuged for 5 minutes at 16000 g. To reduce soluble proteins, 6μL of 0.5M dithioerythritol (DTE) was added to each sample vial and incubated at 56°C for 60 minutes. After incubation, 2M ammonium bicarbonate was added to each sample vial until the pH of the supernatant was 7.5-8.0. Alkylation was performed by adding 12μL of 0.5M iodoacetamide to the sample vials and incubated in the dark at 25°C for 60 minutes. The carbamidomethylation reaction was quenched by adding 12μL of 0.5M DTE to the sample vials and incubated at room temperature for 5 minutes. After incubation, 0.01% Protease Max (Promega Inc.) was added to the sample vials along with 0.5μg mass spectrometry grade trypsin (Thermo Pierce Inc.). Each sample was incubated at room temperature for 20 hours at 300 rpm. After incubation, sample vials were centrifuged for 5 minutes and 200μL of the supernatant transferred to 0.22μm centrifugal filters and centrifuged for 30 minutes. The filtrate was then transferred to clean Eppendorf Protein LoBind tubes for ZipTip (Millipore Inc.) sample clean up to prepare for mass spectrometric analysis. Organic contaminants in aqueous stocks and solutions were removed by prior passage over solid phase extraction (SepPak, C18, Waters Inc.). Blank samples were prepared alongside each batch of samples.

Digested peptides were desalted and concentrated using ZipTip C18 pipette tips (Millipore Inc.) with the eluted material lyophilized and stored at 4°C in Lo-Bind (Eppendorf) tubes until analyzed via liquid chromatography-tandem mass spectrometry. Prior to analysis, samples were resuspended in 2% (v/v) acetonitrile and 0.1% (v/v) TFA. Peptide concentration was measured using the Pierce Quantitative Fluorometric Peptide Assay (Thermo Pierce, Inc.) and 1 µg of peptide, or 40% of the total sample if the sample was too diluted, was applied to mass spectrometry.

Liquid chromatography-tandem mass spectrometry of peptides was accomplished with a Thermo Scientific Q-Exactive Plus Orbitrap mass spectrometer equipped with a Proxeon Easy-nLC II HPLC (Thermo Scientific). Samples were loaded a 100 micron x 25 mm Magic C18 100Å 5U reverse phase trap where they were desalted online before being separated using a 75 micron x 150 mm Magic C18 200Å 3U reverse phase column. Peptides were eluted using a 65 minute gradient with a flow rate of 300nl/min. An MS survey scan was obtained for the m/z range 300-1600, MS/MS spectra were acquired using an inclusion list of 28 ions (Parker et al. 2019) that were subjected to HCD (High Energy Collisional Dissociation). When inclusion list ions were not found, MS/MS was done on other ions in the MS survey scan. An isolation mass window of 1.6 m/z was used for precursor ion selection, and normalized collision energy of 27% used for fragmentation. A five second duration was used for the dynamic exclusion. Washes were applied between each sample. After 10 samples a blank run of BSA standards was applied to test for sample-to-sample contamination.

**Proteomic Data Processing**

Mass spectrometry datasets (.RAW format) were processed with PEAKS (10.0) peptide matching software (Bioinformatics Solutions Inc., Waterloo, ON). The FASTA formatted UNIPROT *Homo sapiens* reference protein database (<http://www.uniprot.org/proteomes/UP000005640>) was modified to include additional FASTA protein entries of peptide sequences from all splice variants associated with AMELX_HUMAN (Q99217-1, -2, -3) and AMELY_HUMAN (Q99218-1, -2) proteins gene products (Salido, et al., 1992, Simmer, 1995). The reference database was further modified to incorporate a decoy database and was validated in PEAKS Software (Zhang, et al., 2012). Peptide matching spectral assignment was conducted using default conditions with the following exceptions: error tolerance was set to 10 ppm for precursor mass and 0.04Da for fragment ions; cleavage with trypsin was set to non-specific, and up to two missed cleavages. The algorithm assumed all cysteines were carbamidomethylated, and the peptide was partially modified by deamidation (NQ), oxidation (MHW) and dioxidation (M), and pyroglutamate conversion from glutamate and glutamine. All peptide assignments were filtered by a 1% false discovery rate (Zhang et al. 2012). Each peptide was quantified by using the peak ion intensity of the primary precursor mass over charge ratio (m/z). Peptides composed entirely of paralogous amino acid sequences were further filtered from results. Maximum ion intensity signals from all peptides specific to either the AMELY_HUMAN or AMELX_HUMAN gene product were then combined into a cumulative single metric (combined intensities, CI), normalized for total enamel sampled (mg), and corrected for the proportion of the sample applied to the instrument (CI per mg enamel). Detection of peptides specific to the AMELY_HUMAN gene product was considered unambiguous evidence of male sex. In the absence of AMELY_HUMAN peptides, female probability, Pr(F), was calculated as a function of the combined intensity of AMELX peptides, based on logistic regression of known standards where increased AMELX signal increased the probability of female sex (Parker et al. 2019). Specifically, AMELX_HUMAN signals (CI/mg) were log transformed and then solved for Pr(F) using the equation Pr(F) = 1.0 + (0.059-1.0)/(1+(x/7.54)^13.99^ where “x” is the logarithm (base 10) of the AMELX_HUMAN (Parker et al. 2019). Samples with a Pr(F) < 0.5 were considered indeterminate for proteomic sex estimation. The mass spectrometry proteomics data, along with customized protein reference library, have been deposited to the ProteomeXchange Consortium via the PRIDE partner repository with the accession number PXD016076 (http://www.proteomexchange.org)(Deutsch, et al., 2017).

**Consistency of Amelogenin Quantification**

Given the relative novelty of the proteomic technique, several duplicate samples from the same individual were prepared and analyzed to check consistency. Duplicate proteomic samples were also extracted from the teeth of individuals including two cases where there was a disagreement with DNA (ALA-565/H, B47 and B62), and another case where DNA failed for reconstruction (ALA-565/H, B30, a cremation). All proteomic duplicates agreed with original proteomic sex assignments except for one, ALA-565/H-B62. Burial 62 originally had an indeterminate sex assignment based no AMELY peptides but a Pr(F)= 0.29. A second analysis had a higher amount of AMELX peptides, but still no AMELY peptides, giving it a Pr(F) of 0.68 and changing this assignment to female. This is consistent with definitive DNA-based female sex estimates for the same sample. Data for all duplicate samples, along with reagent blank runs, are provided in supplemental material (Tables S2 and S3).

Burial 62 illustrates a potential problem with assigning male sex in the absence of male peptides as originally described in Parker et al. (2019). Given the possibility of very poor conditions for preservation in some archaeological contexts, we cannot rule out the possibility of a female sample with very low amounts of AMELX. In light of this, we recommend a slight refinement by designating samples with Pr(F) values below 0.50 as indeterminate, while samples above 0.50 are assigned female at their respective probabilities.

**Regression analysis of AMELX signal (log transformed) versus number of DNA reads (log transformed) for all matched samples from *Síi Túupentak* (CA-ALA-565/H) and *Rummey Ta Kuččuwiš Tiprectak (*CA-ALA-704/H)**

Summary statistics:

| Variable | Observations | Obs. with missing data | Obs. without missing data | Minimum | Maximum | Mean | Std. deviation |
| --- | --- | --- | --- | --- | --- | --- | --- |
| AMELX (CI) | 53 | 0 | 53 | 7.780 | 10.028 | 9.060 | 0.581 |
| Total Reads | 53 | 0 | 53 | 2.305 | 7.593 | 4.796 | 1.250 |

Regression of variable AMELX (CI):

Goodness of fit statistics (AMELX (CI)):

| Observations | 53.000 |
| --- | --- |
| Sum of weights | 53.000 |
| DF | 51.000 |
| R² | 0.056 |
| Adjusted R² | 0.037 |
| MSE | 0.325 |
| RMSE | 0.570 |
| MAPE | 4.920 |
| DW | 2.256 |
| Cp | 2.000 |
| AIC | -57.588 |
| SBC | -53.648 |
| PC | 1.018 |

Analysis of variance (AMELX (CI)):

| Source | DF | Sum of squares | Mean squares | F | Pr > F |
| --- | --- | --- | --- | --- | --- |
| Model | 1 | 0.982 | 0.982 | 3.021 | 0.088 |
| Error | 51 | 16.581 | 0.325 |  |  |
| Corrected Total | 52 | 17.563 |  |  |  |
| *Computed against model Y=Mean(Y)* | | | |  |  |

Model parameters (AMELX (CI)):

| Source | Value | Standard error | t | Pr > \|t\| | Lower bound (95%) | Upper bound (95%) |
| --- | --- | --- | --- | --- | --- | --- |
| Intercept | 9.587 | 0.313 | 30.605 | **< 0.0001** | 8.958 | 10.216 |
| Total Reads | -0.110 | 0.063 | -1.738 | 0.088 | -0.237 | 0.017 |

Equation of the model (AMELX (CI)):

AMELX (CI) = 9.58725211280958-0.109929304082137*Total Reads

Figure: Regression of AMELX (CI) by Total Reads (R²=0.056)

**Independent t-test for average AMELX CI/mg signal (log transformed) recovered from samples at *Síi Túupentak* (CA-ALA-565/H) and *Rummey Ta Kuččuwiš Tiprectak (*CA-ALA-704/H)**

Summary statistics:

| Variable | Observations | Obs. with missing data | Obs. without missing data | Minimum | Maximum | Mean | Std. deviation |
| --- | --- | --- | --- | --- | --- | --- | --- |
| Var1 | 39 | 0 | 39 | 7.871 | 10.012 | 9.031 | 0.564 |
| Var1 | 16 | 0 | 16 | 7.780 | 10.028 | 9.081 | 0.630 |

t-test for two independent samples / Two-tailed test:

95% confidence interval on the difference between the means:

[ -0.397,0.298 ]

| Difference | -0.049 |
| --- | --- |
| t (Observed value) | -0.284 |
| \|t\| (Critical value) | 2.006 |
| DF | 53 |
| p-value (Two-tailed) | 0.777 |
| alpha | 0.05 |


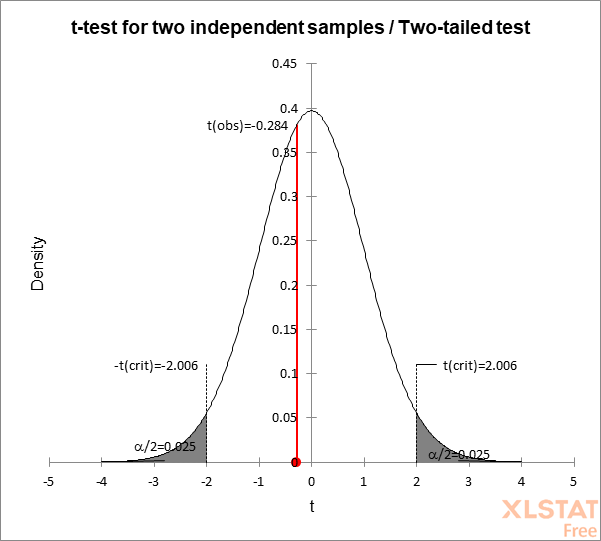


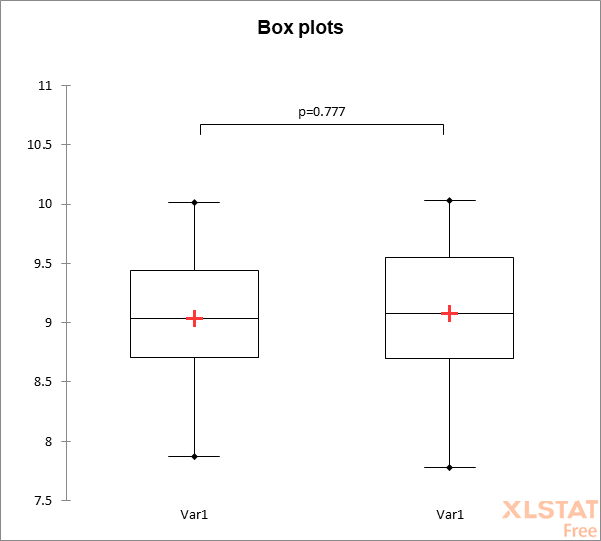


**Independent t-test for average n DNA seq (log transformed) recovered from samples at *Síi Túupentak* (CA-ALA-565/H) and *Rummey Ta Kuččuwiš Tiprectak (*CA-ALA-704/H)**

Summary statistics:

| Variable | Observations | Obs. with missing data | Obs. without missing data | Minimum | Maximum | Mean | Std. deviation |
| --- | --- | --- | --- | --- | --- | --- | --- |
| Var1 | 39 | 2 | 37 | 3.584 | 7.593 | 5.134 | 1.102 |
| Var1 | 16 | 0 | 16 | 2.305 | 5.984 | 4.015 | 1.254 |

t-test for two independent samples / Two-tailed test:

95% confidence interval on the difference between the means:

[ 0.429,1.809 ]

| Difference | 1.119 |
| --- | --- |
| t (Observed value) | 3.257 |
| \|t\| (Critical value) | 2.008 |
| DF | 51 |
| p-value (Two-tailed) | 0.002 |
| alpha | 0.05 |


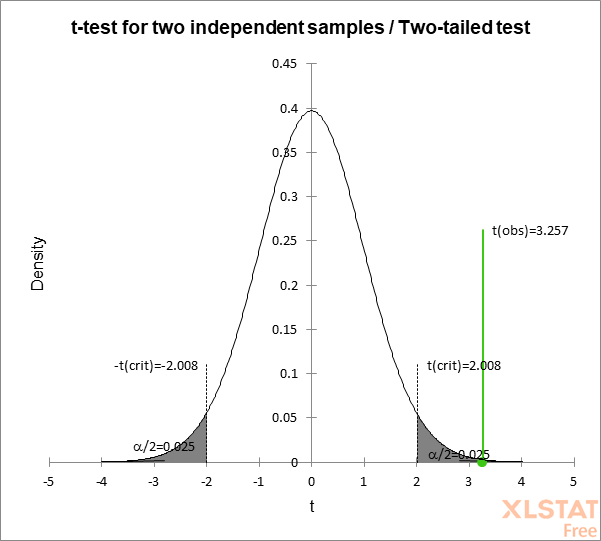


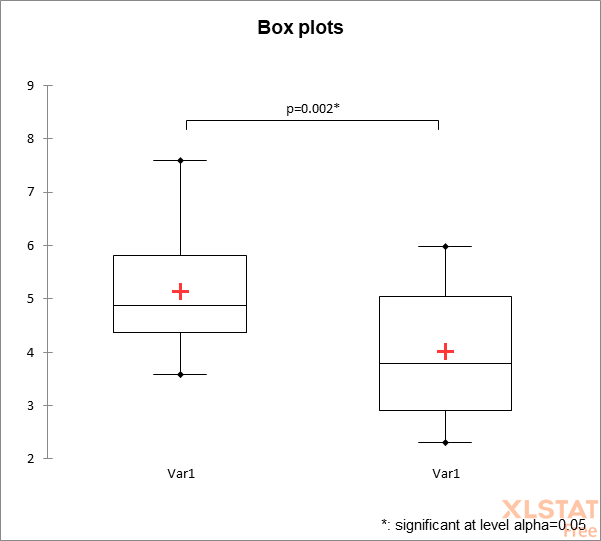

Supplement: Supplementary file 1 — Supplementary file [file 41598_2020_68550_MOESM1_ESM.docx]
